# Supplementary material for: Sorting at embryonic boundaries requires high heterotypic interfacial tension
Source: Nat Commun. 2017 Jul 31;8:157. doi: 10.1038/s41467-017-00146-x (PMC5537356; doi:10.1038/s41467-017-00146-x)
Supplement: Supplementary file 2 — Supplementary Software 1 [file 41467_2017_146_MOESM2_ESM.zip › PottsModel/SrcPottsModel/doc/model/Pixel.html]

Pixel


JavaScript is disabled on your browser.


Skip navigation links


- Overview
- Package
- Class
- Use
- Tree
- Deprecated
- Index
- Help

- Prev Class
- Next Class

- Frames
- No Frames

- All Classes

- Summary:
- Nested |
- Field |
- Constr |
- Method

- Detail:
- Field |
- Constr |
- Method


model

## Class Pixel

- java.lang.Object
- - model.Pixel

- All Implemented Interfaces:
  :   KnowsConstants

  ---

    

  ```
  public class Pixel
  extends java.lang.Object
  implements KnowsConstants
  ```

- - ### Nested Class Summary

    Nested Classes

    | Modifier and Type | Class and Description |
    | --- | --- |
    | `static class` | `Pixel.PixelType` |
  - ### Field Summary

    Fields

    | Modifier and Type | Field and Description |
    | --- | --- |
    | `int` | `aX` |
    | `int` | `aY` |
  - ### Constructor Summary

    Constructors

    Constructor and Description

    `Pixel(int pX,
    int pY,
    Cell pC,
    Lattice pL)`

    `Pixel(Pixel pPixel)`

    `Pixel(Pixel pPixel,
    Cell pC)`
  - ### Method Summary

    All Methods Static Methods Instance Methods Concrete Methods

    | Modifier and Type | Method and Description |
    | --- | --- |
    | `boolean` | `adjustedBoundary()` Checks whether this APixel is at a cell boundary i.e. |
    | `static boolean` | `areSwappable(Pixel pSource, Pixel pTarget)` Check if resulting swap will not produce a disconnected pixel. |
    | `boolean` | `atLatticeEdge()` |
    | `Pixel[]` | `copyTo(Pixel pTarget)` Move pixel from one cell to the other and make appropriate changes. |
    | `boolean` | `equals(java.lang.Object pOtherPixel)` |
    | `Pixel[]` | `getAllNeighbors()` |
    | `static double` | `getAreaUnit(PixelShape.Type pType)` |
    | `Cell` | `getCell()` |
    | `boolean[]` | `getCellBoundaryEdges()` Get array of active edges (true) in the same order as getValidEdges() defined in PixelShape. |
    | `Cell.CellType` | `getCellType()` |
    | `java.awt.Color` | `getColor()` |
    | `Pixel[]` | `getConnectedNeighbors()` |
    | `Constants` | `getConstants()` |
    | `Coordinates` | `getCoordinates()` |
    | `static Pixel.PixelType` | `getDefaultPixelType(Cell.CellType pCellType)` |
    | `PixelDisplay` | `getDisplay()` |
    | `PixelDisplay` | `getDisplay(int pSize)` |
    | `java.awt.Point` | `getDisplayCenterPoint()` |
    | `static double` | `getEdgeUnit(PixelShape.Type pType)` |
    | `java.lang.String` | `getInformation()` |
    | `double` | `getLocalTemperature()` |
    | `int` | `getNumberOfNeighborsFromCell(Cell aCell)` |
    | `static int` | `getNumPixelEdges(PixelShape.Type pType)` |
    | `double` | `getPixelInteraction(Pixel pNeighbor)` Checks whether pixels are neighbors and then calculates interaction based on cell type. |
    | `static double` | `getPixelInteraction(Pixel pPixel1, Pixel pPixel2)` Checks whether pixels are neighbors and then calculates interaction based on cell type. |
    | `IPositionManager` | `getPositionManager()` |
    | `Pixel` | `getRandomNeighbor(java.util.Random r)` |
    | `Pixel.PixelType` | `getType()` |
    | `int` | `getX()` |
    | `int` | `getY()` |
    | `int` | `hashCode()` |
    | `void` | `initialize()` |
    | `boolean` | `isBoundary()` |
    | `boolean` | `isInSameCell(Pixel pTarget)` |
    | `boolean` | `isMedium()` |
    | `boolean` | `isNeighborWith(Pixel pPixel)` |
    | `java.lang.String` | `parametersToString()` |
    | `java.lang.String` | `toString()` |

    - ### Methods inherited from class java.lang.Object

      `getClass, notify, notifyAll, wait, wait, wait`

- - ### Field Detail


    - #### aX

      ```
      public final int aX
      ```


    - #### aY

      ```
      public final int aY
      ```
  - ### Constructor Detail


    - #### Pixel

      ```
      public Pixel(int pX,
                   int pY,
                   Cell pC,
                   Lattice pL)
      ```


    - #### Pixel

      ```
      public Pixel(Pixel pPixel,
                   Cell pC)
      ```


    - #### Pixel

      ```
      public Pixel(Pixel pPixel)
      ```
  - ### Method Detail


    - #### parametersToString

      ```
      public java.lang.String parametersToString()
      ```


    - #### initialize

      ```
      public void initialize()
      ```


    - #### atLatticeEdge

      ```
      public boolean atLatticeEdge()
      ```


    - #### areSwappable

      ```
      public static boolean areSwappable(Pixel pSource,
                                         Pixel pTarget)
      ```

      Check if resulting swap will not produce a disconnected pixel.

      Parameters:
      :   `pPixel1` -
      :   `pPixel2` -

      Returns:


    - #### copyTo

      ```
      public Pixel[] copyTo(Pixel pTarget)
      ```

      Move pixel from one cell to the other and make appropriate changes.

      Parameters:
      :   `pCell` -


    - #### getColor

      ```
      public java.awt.Color getColor()
      ```


    - #### getType

      ```
      public Pixel.PixelType getType()
      ```


    - #### getDisplayCenterPoint

      ```
      public java.awt.Point getDisplayCenterPoint()
      ```


    - #### getDefaultPixelType

      ```
      public static Pixel.PixelType getDefaultPixelType(Cell.CellType pCellType)
      ```


    - #### getCell

      ```
      public Cell getCell()
      ```


    - #### getCellType

      ```
      public Cell.CellType getCellType()
      ```


    - #### getNumberOfNeighborsFromCell

      ```
      public int getNumberOfNeighborsFromCell(Cell aCell)
      ```


    - #### getAllNeighbors

      ```
      public Pixel[] getAllNeighbors()
      ```


    - #### getConnectedNeighbors

      ```
      public Pixel[] getConnectedNeighbors()
      ```


    - #### getRandomNeighbor

      ```
      public Pixel getRandomNeighbor(java.util.Random r)
      ```


    - #### toString

      ```
      public java.lang.String toString()
      ```

      Overrides:
      :   `toString` in class `java.lang.Object`


    - #### getInformation

      ```
      public java.lang.String getInformation()
      ```


    - #### getLocalTemperature

      ```
      public double getLocalTemperature()
      ```


    - #### getPixelInteraction

      ```
      public double getPixelInteraction(Pixel pNeighbor)
      ```

      Checks whether pixels are neighbors and then calculates interaction based
      on cell type. Interactions include: \* within cell \* meso-medium \*
      ecto-medium \* meso-ecto \* meso-meso \* ecto-ecto TODO: improve flexibility

      Returns:
      :   energy of the interaction


    - #### getPixelInteraction

      ```
      public static double getPixelInteraction(Pixel pPixel1,
                                               Pixel pPixel2)
      ```

      Checks whether pixels are neighbors and then calculates interaction based
      on cell type. Interactions include: \* within cell \* meso-medium \*
      ecto-medium \* meso-ecto \* meso-meso \* ecto-ecto TODO: improve flexibility

      Returns:
      :   energy of the interaction


    - #### isInSameCell

      ```
      public boolean isInSameCell(Pixel pTarget)
      ```


    - #### isNeighborWith

      ```
      public boolean isNeighborWith(Pixel pPixel)
      ```


    - #### adjustedBoundary

      ```
      public boolean adjustedBoundary()
      ```

      Checks whether this APixel is at a cell boundary i.e. whether it has at
      least one neighbor with a different cell ID.

      Parameters:
      :   `isBoundary` - true if this APixel is at a cell boundary

      Returns:


    - #### isBoundary

      ```
      public boolean isBoundary()
      ```


    - #### getCoordinates

      ```
      public Coordinates getCoordinates()
      ```


    - #### getX

      ```
      public int getX()
      ```


    - #### getY

      ```
      public int getY()
      ```


    - #### getDisplay

      ```
      public PixelDisplay getDisplay()
      ```


    - #### getDisplay

      ```
      public PixelDisplay getDisplay(int pSize)
      ```


    - #### hashCode

      ```
      public int hashCode()
      ```

      Overrides:
      :   `hashCode` in class `java.lang.Object`


    - #### equals

      ```
      public boolean equals(java.lang.Object pOtherPixel)
      ```

      Overrides:
      :   `equals` in class `java.lang.Object`


    - #### getCellBoundaryEdges

      ```
      public boolean[] getCellBoundaryEdges()
      ```

      Get array of active edges (true) in the same order as getValidEdges()
      defined in PixelShape.

      Returns:
      :   boolean array where true means that corresponding Edge is a cell
          boundary

      See Also:
      :   `PixelShape`


    - #### isMedium

      ```
      public boolean isMedium()
      ```


    - #### getEdgeUnit

      ```
      public static double getEdgeUnit(PixelShape.Type pType)
      ```


    - #### getAreaUnit

      ```
      public static double getAreaUnit(PixelShape.Type pType)
      ```


    - #### getNumPixelEdges

      ```
      public static int getNumPixelEdges(PixelShape.Type pType)
      ```


    - #### getPositionManager

      ```
      public IPositionManager getPositionManager()
      ```


    - #### getConstants

      ```
      public Constants getConstants()
      ```

      Specified by:
      :   `getConstants` in interface `KnowsConstants`


Skip navigation links


- Overview
- Package
- Class
- Use
- Tree
- Deprecated
- Index
- Help

- Prev Class
- Next Class

- Frames
- No Frames

- All Classes

- Summary:
- Nested |
- Field |
- Constr |
- Method

- Detail:
- Field |
- Constr |
- Method
